# Supplementary figures and images for: PKR activation-induced mitochondrial dysfunction in HIV-transgenic mice with nephropathy
Source: eLife. 2024 Aug 29;12:RP91260. doi: 10.7554/eLife.91260 (PMC11361708; doi:10.7554/eLife.91260)

Raw blot images for Figure3D

|      |      |     |      |
|------|------|-----|------|
| WT   | Tg26 | WT  | Tg26 |
| Cont |      | C16 |      |

IB:OXPHOS

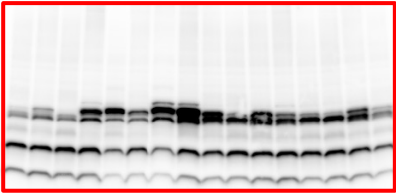

IB:VDAC

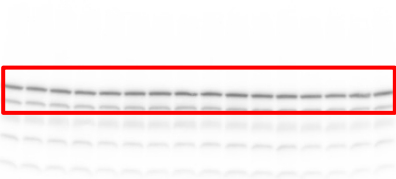

Supplement: Figure 3—source data 1. [file elife-91260-fig3-data1.zip › Figure 3-source data1/Figure 3D-Source data 1 Uncropped and labeled gels for Figure 3D.pdf.pdf]

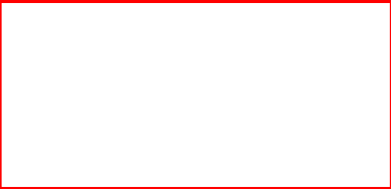

Supplement: Figure 3—source data 2. [file elife-91260-fig3-data2.zip › Figure3-source data2/Figure 3D-source data 1 Raw unedited gels for Figure 3D.pdf]

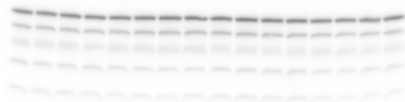

Supplement: Figure 3—source data 2. [file elife-91260-fig3-data2.zip › Figure3-source data2/Figure 3D-source data 2 Raw unedited gels for Figure 3D.pdf]

Raw blot images for Figure 5C

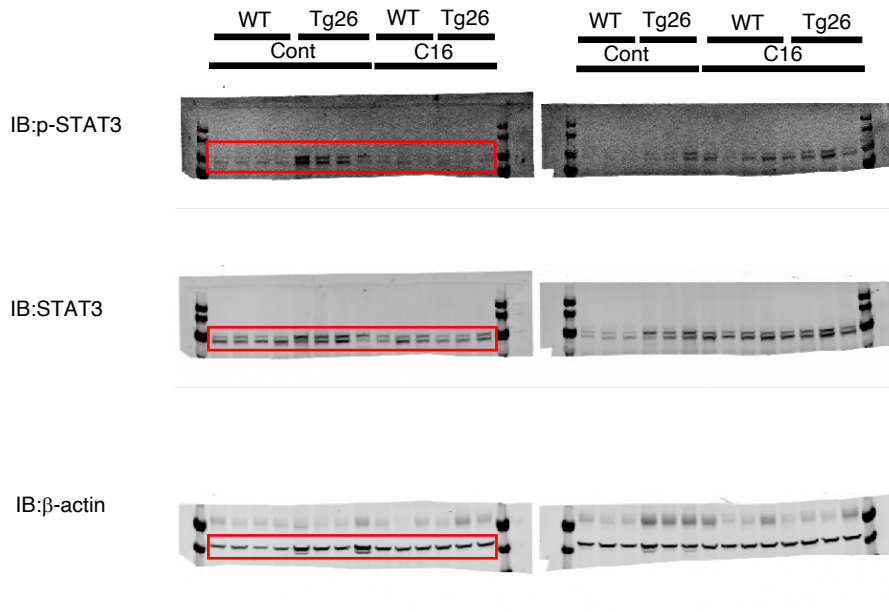

Supplement: Figure 6—source data 1. [file elife-91260-fig6-data1.zip › Figure6-source data1/Figure 5C-Source data 1 Uncropped and labeled gels for Figure 5C.pdf]

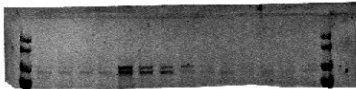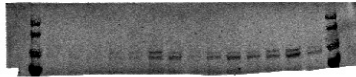

Supplement: Figure 6—source data 2. [file elife-91260-fig6-data2.zip › Figure6-source data2/Figure 5C-source data 1 Raw unedited gels for Figure 5C.pdf]

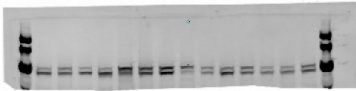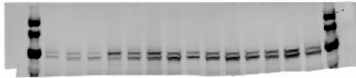

Supplement: Figure 6—source data 2. [file elife-91260-fig6-data2.zip › Figure6-source data2/Figure 5C-source data 2 Raw unedited gels for Figure 5C.pdf]

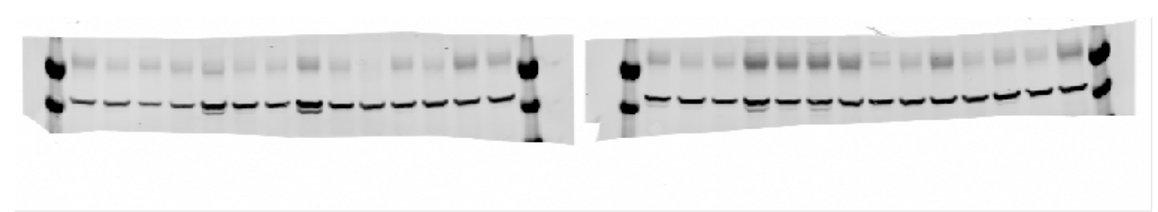

Supplement: Figure 6—source data 2. [file elife-91260-fig6-data2.zip › Figure6-source data2/Figure 5C-source data 3 Raw unedited gels for Figure 5C.png]
